# Uncommon Subtypes of Malignant Melanomas: A Review Based on Clinical and Molecular Perspectives

**DOI:** 10.3390/cancers12092362

**Published:** 2020-08-21

**Authors:** Matías Chacón, Yanina Pfluger, Martín Angel, Federico Waisberg, Diego Enrico

**Affiliations:** Department of Medical Oncology, Alexander Fleming Cancer Institute, Buenos Aires 1426, Argentina; ypfluger@alexanderfleming.org (Y.P.); mangel@alexanderfleming.org (M.A.); fwaisberg@alexanderfleming.org (F.W.); denrico@alexanderfleming.org (D.E.)

**Keywords:** rare melanomas, uncommon melanomas, targeted therapy, immunotherapy, mucosal melanoma, uveal melanoma, amelanotic melanoma, desmoplastic melanoma, spitzoid melanoma, acral melanoma

## Abstract

Malignant melanoma represents the most aggressive type of skin cancer. Modern therapies, including targeted agents and immune checkpoint inhibitors, have changed the dismal prognosis that characterized this disease. However, most evidence was obtained by studying patients with frequent subtypes of cutaneous melanoma (CM). Consequently, there is an emerging need to understand the molecular basis and treatment approaches for unusual melanoma subtypes. Even a standardized definition of infrequent or rare melanoma is not clearly established. For that reason, we reviewed this challenging topic considering clinical and molecular perspectives, including uncommon CMs—not associated with classical V600E/K *BRAF* mutations—malignant mucosal and uveal melanomas, and some unusual independent entities, such as amelanotic, desmoplastic, or spitzoid melanomas. Finally, we collected information regarding melanomas from non-traditional primary sites, which emerge from locations as unique as meninges, dermis, lymph nodes, the esophagus, and breasts. The aim of this review is to summarize and highlight the main scientific evidence regarding rare melanomas, with a particular focus on treatment perspectives.

## 1. Introduction

Malignant melanoma is one of the most aggressive cancers once it becomes metastatic, thus an early identification has a high impact on prognoses. In less than ten years, melanoma has become a successful model where preclinical and clinical advances in research could provide meaningful improvements on patients’ survival and quality of life. Melanoma was one of the first tumor models where targeted agents and immunotherapy have revolutionized patient outcomes. However, most of this scientific progress focused mainly on studying patients with cutaneous melanoma (CM), representing the most common subtype.

Rare melanoma variants usually account for less than 5% of all melanomas, and often are associated with a poor prognosis [1,2,3]. Of note, the molecular basis and treatment approaches for patients with unusual melanomas are still not elucidated. Furthermore, a uniform definition of these “rare melanomas” has not been clearly established. Our review provides an overview of the clinical, biological, and mutational landscapes of rare melanoma subtypes, summarizing the most relevant evidence on therapeutic approaches. 

## 2. Cutaneous Melanoma

### 2.1. Introduction

CM represents the most lethal and frequent type of skin cancer. Between 40% and 60% of CMs harbor activating *BRAF* mutations, characterized by the substitution of the valine residue at position 600 by glutamate (V600E) or lysine (V600K), representing 70–90% and 10–20% of somatic alterations of this gene, respectively [4,5,6,7]. The determination of molecular predictive factors has become essential for treatment definitions in patients diagnosed with stage III or IV CM. A dual MEK/BRAF blockade in patients harboring *BRAF* V600E/K mutant CMs has resulted in significant improvements in the overall survival (OS) in the adjuvant and advanced settings [8,9]. Immune checkpoint inhibitors have also been established as an effective treatment for CM, showing significant increases in recurrence-free survival, progression-free survival (PFS) and the OS in the same scenarios [10,11,12]. In pivotal trials, these results were evident in all biomarker-oriented analyzed subgroups, regardless of mutational status.

In light of current evidence, immune checkpoint inhibitors are considered a standard treatment for patients with CM, and BRAF/MEK inhibitors are recommended in patients with melanoma and BRAF V600E/K mutations [10,11,12].

**Figure 1:**
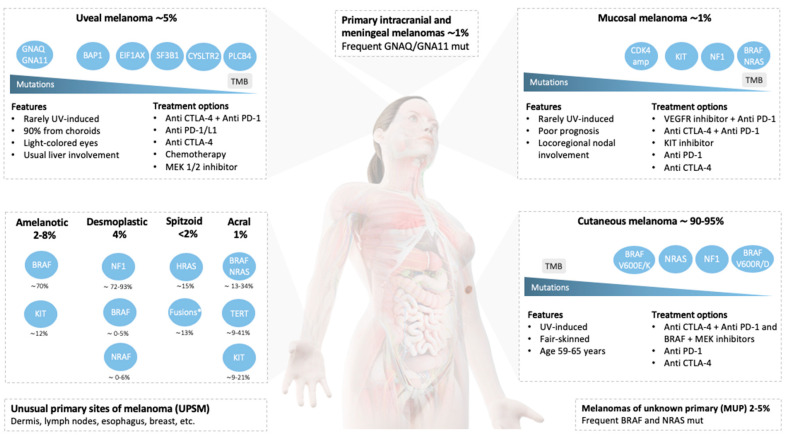
Schematic summary of the most relevant mutations, key features, and treatment options of rare melanomas. For each subtype, mutations are ordered by their prevalence. Main treatment strategies were ordered according to the decreasing efficacy outcomes, including the overall response rate, progression-free survival, and overall survival. Abbreviations: TMB, tumor mutational burden; UV, ultraviolet radiation; Mut, mutation; Amp, amplification; KIT, receptor tyrosine kinase (c-Kit); VEGFR, vascular endothelial growth factor receptor. *Fusion kinases involving ALK, ROS1, NTRK1, NTRK3, MET, RET, BRAF, and MAP3K8.

In the following section, we will summarize CM´s clinical characteristics and treatment approaches not associated with specific therapeutic strategies. First, we will review current evidence of biomarker-oriented subgroups, considering CM´s genomic classification defined by The Cancer Genome Atlas Network (NRAS, BRAF, NF1, triple wild-type subgroups) [13]. Tumors with BRAF V600E/K mutations will not be addressed in our review due to the fact that treatment strategies are based on phase 3 clinical trials that had evaluated this population. Secondly, we will describe the clinical features and treatment approaches in special morphological entities, such as amelanotic, desmoplastic, spitzoid, and acral melanomas. All these entities were underrepresented in pivotal practice-changing trials. In each subsection, clinical characteristics and relevant published studies will be addressed. (Figure 1).

### 2.2. Genetic Landscape and Specific Treatment Approaches

#### 2.2.1. NRAS

*NRAS* gene mutations contribute to the activation of mitogen-activated protein kinase (MAPK) pathway signaling, inducing melanocytogenesis, and increasing cell proliferation and survival. It is estimated that up to 25% of CMs harbor *NRAS* gene alterations, 80% of these being Q61R, Q61K, and Q61L point mutations [14].

Primary lesions in this subtype are associated with clinical features such as ulceration, high levels of Breslow depth, an increased mitotic rate, and chronic ultraviolet exposure [15]. Particularly, *NRAS* mutations were found in 21% of superficial spreading, 31% of nodular and 8% of acral melanoma subtypes, and they are frequent among patients older than 55 years [16,17]. Of note, advanced NRAS-mutant diseases have been associated with central nervous system involvement at diagnosis [14]. The response rates and duration of responses for targeted therapy in patients with *NRAS*-mutant melanoma have proven to be modest. Selected studies are described in Table 1.

In the advanced setting, immune checkpoint inhibitors, such as monotherapy or in combination, remain the main therapeutic strategies. Kirchberger and collaborators retrospectively analyzed 364 patients with advanced melanoma who received immunotherapy. The authors found that the response rates among patients with and without *NRAS* mutations were similar (31% vs. 26% for the anti-PD-1/anti-CTLA-4 combination and 21% vs. 13% for the anti-PD-1 monotherapy, respectively) [27]. Interestingly, a better response rate with immunotherapy combination was observed in patients with Q61L *NRAS* mutations. A trend of superior PFS was also evidenced in subjects with a Q61L NRAS-mutant disease in another retrospective analysis [28]. With the current evidence, the combination of immune checkpoint inhibitors is the common first treatment approach for patients with tumors harboring this mutation.

Ongoing clinical trials are evaluating novel MEK inhibitors (FCN-159), pan-RAF therapies (belvarafenib), a combination of MEK inhibitors and therapies that target other relevant mechanisms, such as autophagy (hydroxychloroquine) and immune checkpoint inhibitors [22,29,30,31]. Areas of further investigation include the inhibition of enzymes that are overexpressed in NRAS-mutant melanoma, including polo-like kinase, ROCK1/2, and Tand-binding kinase 1 [32]. Moreover, Dinter and collaborators have suggested a potential role of the combination of MEK and BRAF inhibitors, due to the increased levels of endoplasmic reticulum stress detected in NRAS-mutant melanoma cell lines [33].

#### 2.2.2. NF1

Representing around 15% of CM cases, *NF1* loss leads to decreased RAS-GTP dephosphorylation, resulting in an increase in RAS-GTP and subsequently RAF-MEK-ERK phosphorylation [13].

*NF1* mutations are particularly frequent in patients of an older age. Additionally, the associated clinical features include chronic sun exposure, desmoplastic melanoma, UV mutational signatures, and a high tumoral mutation burden [34]. Notably, in patients with neurofibromatosis, type 1 melanoma risk is only increased by 3.6-fold [35].

Importantly, Garman and collaborators, after characterizing 30 *NF1* mutant tumor biopsies, patient-derived xenografts and cell lines, documented the coexistence of non-V600E *BRAF*, *RAS,* and other characteristic MAP kinase-associated genes mutations in 87% of the analyzed cases. This finding is in accordance with functional studies that documented that not all NF1 mutant cell lines were sensitive to MEK inhibitors [36]. Therefore, a further characterization of the NF1 mutant subgroup is needed, considering the occurrence of concurrent mutations.

Immunotherapy remains the main strategy for this subgroup of patients. In a study performed by Eroglu et al., among 17 evaluable patients with desmoplastic melanoma, 14 harbored *NF1* alterations. Considering the high overall response rate (ORR) observed in this histologic subgroup, a benefit of checkpoint inhibitors is expected in patients with CM and *NF1* mutations [37] and is the principal treatment option for this population.

#### 2.2.3. Uncommon BRAF Mutations

Pivotal studies that determined the approval of available target combinations did not include subjects with gene alterations, apart from V600E and V600K [9,38]. Consequently, the therapeutic implications in this population have mostly been assessed in retrospective studies.

V600R represents 5–7% of *BRAF* mutations, constituting the third most frequent alteration. Comparable to patients with melanoma and V600K mutations, V600R alterations are more prevalent in men and older patients [39]. Regular characteristics include tumor ulceration, primary localizations associated with cumulative sun-induced DNA damage, and short disease-free intervals between the primary diagnosis and the occurrence of distant lesions [40,41]. Menzer and colleagues described the results of the largest multicenter retrospective study that included patients with uncommon *BRAF* mutations [42]. Notably, among 26 patients with *BRAF* V600R mutations, a significant improvement in the median OS (22.9 vs. 7.3 months, *p* = 0.002), median PFS (8.0 vs. 3.8 months, *p* = 0.002) and ORR (57 vs. 22%) was observed when MEK inhibitors were administrated together with BRAF inhibitors in comparison to the group that was only assigned to the treatment with BRAF inhibitors. Less information is available regarding other *BRAF* codon 600 mutations. According to Menzer and colleagues’ study, a tumoral response with BRAF or BRAF/MEK inhibition was observed in patients that harbored *BRAF* V600D (four of five) and V600M mutations (one of two). All seven cases were associated with clinical benefits. Although further study is needed, these results support that BRAF and MEK inhibition is an effective strategy for patients with melanoma and uncommon codon 600 BRAF mutations.

Unlike non-small cell lung cancer, *BRAF* mutations that do not affect codon 600 (non-600) are particularly infrequent in melanoma. A higher prevalence of this alteration is observed in patients with head and neck melanomas.

In this group, mutations could be characterized regarding the kinase activity. In a recent report published by Lokhandwala and collaborators, class II mutations (RAS-independent kinase-activating dimers not involving codon 600), such as L597Q/R/S, K601E and G469A/R/V, represented 7.4% of cases with *BRAF* mutations. Class III mutations (associated with a low BRAF activity) were observed in 12% of *BRAF*-mutant patients. Examples of the latter include G466A/E/V, S467L, N581I, and D594E/G/N [43].

Menzer and colleagues observed that only two out of nine patients with codon 597 and one out of four patients with K601E mutations presented tumoral responses with dual inhibition [42]. Contrastingly, two of the three patients with codon 469 alterations achieved a tumor response.

While the efficacy of target therapies in patients with BRAF class II mutations is still unclear, the National Comprehensive Cancer Network (NCCN) guidelines consider BRAF/MEK inhibition as a recommended strategy for patients with L597 and 601 *BRAF* mutations [44].

#### 2.2.4. Actionable Mutations in the Triple Wild-Type Subgroup (No Mutations in BRAF, RAS, or NF1)

Tumor-agnostic drug approvals offer a window of opportunity for melanoma patients. In this context, kinase fusions, including *ALK*, *RET*, *ROS1*, *BRAF,* and *NTRK*, are characteristic of Spitz melanomas.

Including all CM subtypes, Busam and collaborators have performed immunohistochemistry for *ALK* detection in 603 samples of metastatic and primary tumors [45]. Nine metastatic tumors (3%) and seven primary CMs (2.3%) were classified as ALK-positive. Notably, after performing RNA sequencing, positive samples presented an isoform associated with alternative transcriptional initiation (ATI) sites.

Concomitantly, Lezcano and collaborators have identified among 751 analyzed melanoma samples, four cases with *NTRK* fusions. Interestingly, all four cases presented epitheloid cell figures and were amelanotic [46].

Gene fusions and chromosomic translocations were also evidenced in patients with acral melanoma. For instance, Niu and colleagues have determined the ALK breakpoints in 4 of 28 samples of patients with acral melanoma [47]. Additionally, in the context of the STARTRK-1 trial, a *GOPC-ROS1* fusion was identified in 1 out of 22 patients with this melanoma subtype. This individual was reported to achieve a partial response that lasted at least 11 months with entrectinib. Moreover, a patient with an acral melanoma and a *RET* fusion was reported by Turner and colleagues [48].

KIT signaling plays an essential role in the development of melanocytes, as demonstrated in infrequent genetic disorders associated with hypopigmentation, such as piebaldism. While in *NRAS-* or *BRAF*-mutant melanomas, there are increased levels of *KIT* promoter hypermethylation. It has been classically estimated that around 2–8% of melanomas that arise within cumulative sun-damaged skin exhibit *KIT* gene mutations [49,50]. The efficacy with KIT-directed therapies was modest, as further discussed in the mucosal melanoma section. While immunotherapy is the principal therapeutic approach in these patients, the possibility of defining targetable mutations associated with tumor responses in other cancer models supports the need of further characterization of the “triple wild-type” subgroup.

### 2.3. Entities with Special Morphology

#### 2.3.1. Amelanotic Melanoma

Amelanotic/hypomelanotic melanoma (AM) is a clinicopathological subtype of CM characterized by a decreased or null presence of melanin due to the loss of pigment in tumor evolution, presenting between 2% and 8% of total cases [51]. Due to late recognition, this melanoma subtype is usually diagnosed at more advanced stages, which may explain why patients diagnosed with this entity have a shorter OS compared to CM patients [52]. Other clinical common features of this entity include mostly associations with older age and primary localizations with previous sun exposure, such as the head and neck, trunk, and lower limbs [53]. Interestingly, AM is commonly observed in patients with melanocortin 1 receptor gene (*MC1R*) genotypes linked to particular phenotypes, including red hair color [54,55].

The mechanism underlying amelanosis is still unclear. Although previous studies considered AM as de-differentiated or poorly differentiated melanoma, AM cells maintain the melanocytic lineage and melanin-forming ability, which is demonstrated by tyrosinase and microphthalmia-associated transcription factor (MITF) expression [53,56,57,58]. In this context, AM may result from the insufficient activity of specific melanin formation enzymes, such as tyrosinase [59,60]. Notably, germline mutations in genes for *MC1R*, *MITF*, and *p14ARF* may also result in AM [55,61,62].

The incidence of target mutations in AM has been scarcely characterized. By conducting a sequencing analysis of 33 AM patients, Massi et al. found a *BRAF* V600E and *KIT* mutations rate of 70.3% and 12.1%, respectively [63]. The authors evidenced that *KIT* aberrations were relatively higher in amelanotic lesions in comparison to pigmented primaries.

Considering these findings, *BRAF* mutation analysis in AM may be considered as a potentially valuable diagnostic tool. As with other CM variants, treatment strategies include immune checkpoint inhibitors and the combination of BRAF/MEK inhibitors in the cases where BRAF mutations are present.

#### 2.3.2. Desmoplastic Melanoma

Desmoplastic melanoma (DM) is an uncommon variant (4%) characterized by spindle cells and dense scar-like fibrosis. Histologically, it may resemble other spindle cell lesions of the skin, including spindle cell squamous cell carcinoma, atypical fibroxanthoma, spindle cell sarcoma, and malignant peripheral nerve sheath tumors. Although the S100 stain is usually present, other melanoma markers (HMB-45 and Melan-A) are often negative. SOX10 expression has been shown to be a sensitive and specific marker of DM [64].

Sun exposed areas are commonly affected, and around 60% of lesions are described as non-pigmented, which frequently delays diagnosis [65]. However, DMs are associated with a lower risk of distant metastases in comparison to classic CM [66].

Hotspot mutations in *BRAF* or *NRAS* are not common in DM (0–6%) [34,67]. Though, other alterations in genes related to the MAPK pathway are frequently observed, including *NF1*, *CBL*, *ERBB2*, *MAP2K1*, and *MAP3K1*. Amplifications in *EGFR*, *CDK4*, *MDM2*, *TERT*, *MAP3K1*, *MET*, *NFKBIE*, and *YAP1* are also commonly found in this melanoma subtype [34,67,68].

DM has been associated with UV-induced DNA alterations, presenting a mutation rate four-fold higher than classic CM [67]. Not surprisingly, Boussemart and colleagues found an average tumor mutational burden (TMB) of 77 mut/MB in 12 cases of DM, in comparison to an average TMB of 35 mut/MB obtained after analyzing 1228 samples of other melanoma variants [69]. Consequently, immune checkpoint inhibitors represent a promising strategy in this setting. In a retrospective analysis, Eroglu et al. reported a 70% ORR and 32% complete response rate using anti PD-1/PDL-1 blockade in 60 patients with advanced DM, consolidating checkpoint inhibitors as a key treatment strategy in this particular subgroup [37].

#### 2.3.3. Spitzoid Melanoma

These heterogeneous melanocytic tumors have distinctive histopathologic features, including Spitz nevi, atypical Spitz tumors, and Spitz melanomas. While Spitz tumors are especially frequent in children and adolescents (10–20 years), the incidence of spitzoid melanoma markedly increases in patients older than 20 years [70]. It should be highlighted that Spitz tumors mostly arise in the extremities and face, and lesions arising in other localizations should be carefully examined for a differential diagnosis. Spitz melanomas are characterized by a common regional lymph node spread. In contrast, distant metastases are rarely observed [71,72].

Spitz melanomas, according to the WHO 2018 classification, are defined by the presence of specific genetic hallmarks, such as *HRAS* mutations or fusions in activating genes, including *BRAF*, *NTRK1*, *NTRK3*, *ROS1*, *ALK* and *MAP3K8* [73,74,75]. This characterization has led to distinguishing Spitz melanoma from other spitzoid malignant lesions. In this context, Raghavan and collaborators have documented that only 36% of 25 analyzed spitzoid melanomas were genetically defined as Spitz melanomas [76]. These considerations support that genetic profiling is essential for an accurate diagnostic assessment of this subtype [77].

There is scarce evidence regarding the clinical efficacy of targeted therapy in patients with spitzoid melanomas. A recent presentation highlighted that an 11-year-old patient with this tumor subtype and *MAP3K8* fusion had a non-lasting response with the MEK inhibitor trametinib [78].

Although more information is needed to support specific recommendations, a biomarker-driven approach is reasonable in patients with this melanoma subtype. Treatment possibilities may include BRAF/MEK, ALK (crizotinib, certinib, alectinib), NTRK (entrectnib, larotrectinib) and ROS1 (crizotinib, ceritinib, entrectinib, lorlatinib) inhibitors. In addition, the mechanism of action of farnesyl transferase inhibitors, such as tipifarnib, may represent an interesting approach for patients with *HRAS*-mutant tumors [79].

#### 2.3.4. Acral Lentiginous Melanoma

Acral lentiginous melanoma (ALM) represents only 1% of all melanomas in white populations, exhibiting a higher incidence among Africans, Asians, and descendants of Central Americans [80]. Typical localizations include palms, soles, and nail beds and are frequently characterized by a lentiginous growth pattern. The natural evolution of ALM lesions is slow and it often arises years before diagnosis. Clinical presentation is often observed after foot lesions or associated symptoms, including pain, bleeding, and itching. The advanced stage of the presentation at diagnosis is considered a key contributor to the poor prognosis of this entity.

The frequency of *BRAF* mutations in ALM is estimated to be between 13% and 34% [81,82]. *KIT* mutations and/or amplifications are relatively more common and are present in approximately 9% to 21% of cases. Some phase 2 trials have evaluated the role of KIT inhibitors in this patient subgroup [83]. As described in Table 2, the outcomes in this subgroup were comparable to other melanoma subtypes, and the ORRs evidenced were around 14% to 38%. Other therapeutic approaches are being explored in this rare subtype. Remarkably, 9–41% of ALMs carry activating mutations in *TERT* promoters. Although point mutations cause *TERT* deregulation in UV-exposed melanomas, about 45% of ALMs have *TERT* copy number gains [84]. Telomerase inhibitors have been evaluated in cell lines, and patient-derived xenografts and tumor growth was especially suppressed in cases with *TERT* copy number gains [85]. The obtained results support the further evaluation of telomerase inhibition in patients with ALM.

ALM has been reported to be less susceptible to immune checkpoint inhibitors than other common variants of CM [97]. Explanations of this fact include the low presence of tumor-infiltrating lymphocytes in ALM samples, a low somatic mutational burden, and the lack of a UV-mutational signature [98]. Nevertheless, small retrospective series showed similar tumor response rates when compared to CM [99,100].

Under these circumstances, treatment recommendations in this subgroup include immune checkpoint inhibitors as the main therapeutic strategy. It should be highlighted that the NCCN guidelines describe that KIT inhibitors may be offered in patients with melanoma and activating KIT mutations [44]. As these agents are associated with non-lasting responses, treatment decisions should be carefully addressed on a case by case basis.

## 3. Mucosal Melanoma

### 3.1. Introduction

Mucosal melanoma (MM) is a highly infrequent (~1% of all melanomas) and poor prognosis type of malignant melanoma, arising from melanocytes located in the internal epithelial of different tissues, such as nasopharyngeal, genitourinary, anorectal, and gastrointestinal mucosal membranes. Around half of MMs arise in the head and neck region, followed by the anorectum, and vulva [101].

Particularly, the five-year survival rate is less than 25% in this population, which may be explained by different factors, including the limitation of early visual detection compared to CM, and anatomical factors that hamper a complete resection [102,103]. This uncommon subtype has been considered as a distinctive entity since recent genomic studies have supported the notion that UV-light plays a limited role in carcinogenesis [104,105].

The median age of diagnosis is 70 years, and the incidence of MM is higher in women. MM presents a particular metastatic pattern, most often involving the lungs, liver, and bones [106]. Of note, the locoregional nodal involvement is highly common at diagnosis (>20%) [107]. Justified by the overall poor prognosis for even small superficial lesions, the American Joint Committee on Cancer (AJCC) staging system of head and neck MM only adopted T3 and T4 categories, and the four stages of the disease are represented by III, IVA, IVB, and IVC [108].

A complete surgical excision is the primary treatment strategy for localized MM. However, anatomical limitations hamper the possibility of obtaining wide surgical margins. The role of an adjuvant radiotherapy, chemotherapy, or immunotherapy is still a matter of debate [109,110,111].

Systemic therapy is reserved for patients with advanced or recurrent diseases. Although a precise treatment algorithm cannot be defined for this melanoma subtype, treatment approaches often include immunotherapy as an initial strategy for treating these patients.

### 3.2. Genetic Landscape and Targeted Therapy Approaches

During the last years, whole-exome sequencing and whole-genome sequencing technologies allowed for the characterization of the genetic alterations of MM. Particular alterations in the KIT and MAPK pathways should be specially addressed in this rare tumor due to the fact that they have led to the development of target therapies (Figure 1) [112].

#### 3.2.1. BRAF

*BRAF* mutations are present in MM but at a lower frequency (6–12%) compared to CM [113,114,115]. In a whole-genome sequencing analysis of 67 MM samples performed by Newell et al., *BRAF* mutations were most commonly found in the protein tyrosine kinase domain, with V600E, V600K, and V600R being the most common *BRAF* mutations [116].

On the other hand, non-V600 mutations appear to be present in a higher proportion in MM. In this context, a compiled *BRAF* mutation analysis of 1339 MM performed by Dumaz et al. showed that 37% of mutations were placed on another codon different from V600, particularly on D594 (40%), G469 (24%), and K601 (16%) [113].

No randomized clinical trials have been published on the efficacy and safety of targeted therapy for advanced *BRAF*-mutant MM. In a small cohort of 10 patients with metastatic or unresectable *BRAF* V600E-mutant MM, vemurafenib achieved a 40% ORR and 90% disease control rate (DCR) [117]. In light of these results, and considering the remarkable results of the combination of BRAF and MEK inhibition in CM, these drugs should be considered for *BRAF*-mutant MM. However, despite the initial response, an acquired resistance is expected, and *BRAF* fusions have been proposed as a resistance mechanism to vemurafenib in this population [118].

#### 3.2.2. KIT

The transmembrane tyrosine kinase receptor KIT (v-kit Hardy–Zuckerman 4 feline sarcoma viral oncogene homolog) has a vital role in normal melanocyte growth, differentiation, and migration. Its activation through dimerization regulates multiple downstream signaling pathways, including MAPK and AKT [119].

*KIT* mutations, most commonly in exon 11 and 13, were found at a rate of 13–18% in MM [116]. Of note, *KIT* mutations are especially prevalent in vulvovaginal and anorectal localizations [105,120].

Although Bai et al. have evidenced a worse survival outcome in 66 MM patients with *KIT* mutations, this finding was not replicated by several other studies [121,122,123,124]. Particularly, Hintzsche et al. demonstrated that *NF1* and *KIT* were frequently commutated in 6 out of 19 (32%) MMs [105].

Considering the efficacy of targeted therapy in KIT-addicted tumors, such as gastrointestinal stromal tumors (GISTs), different prospective studies have evaluated KIT inhibition in melanoma. Although drug activity was commonly observed, the median PFS was around 3 to 4 months in most trials (Table 2). A single-group, open-label, phase 2 trial conducted by Carvajal et al., included 28 imatinib mesylate-treated patients with different subtypes of melanomas and *KIT* mutations or amplifications. Among 13 patients (46%) with MM, 23% achieved a clinical response [88]. A multicenter phase 2 trial conducted by Hodi et al. included 17 patients suffering from metastatic MM harboring mutationally activated or amplified *KIT* and treated with imatinib mesylate [89]. Interestingly, the ORR among patients with *KIT* mutations (exon 11, 13, and 17) was 64% (7/11). Contrastingly, imatinib was ineffective in patients that only had *KIT* amplifications since none of the six patients achieved a clinical response. 

Additional phase 2 trials using nilotinib in *KIT*-mutant melanoma patients (including MM) exhibited similar responses as seen with imatinib, demonstrating a clinical effect in patients with disease progression imatinib [90,91,92,93,94]. 

Unlike GISTs, which are characterized by secondary *KIT* gene mutations, the activation of MAPK and PI3K signaling pathways has been proposed as a possible mechanism of resistance in melanoma [125]. Deylon and collaborators have emphasized the role of STAT3 as a key signaling pathway that is inhibited by good responders to nilotinib [94]. These considerations support the development of clinical trials that evaluate KIT inhibitors along with other agents that target different signaling pathways, such as AKT, mTOR, and STAT3 inhibitors.

#### 3.2.3. Others

Other driver mutations are relatively infrequent in MM. The *NRAS* mutation rate is estimated to be around 8% [115]. The most frequent locations affected are similar in both CM and MM (Q61, G12, and G13) [113]. *NRAS* Q61 mutations occur at a lower rate in this population, which may be explained by the association between this particular mutation and UV exposure.

*SPRED1* (sprout related, EVH1 domain-containing protein 1), a negative regulator of the MAPK pathway, was proposed as a tumor suppressor in MM models. *SPRED1* loss is reported to co-occur in 30% of MMs with KIT mutations. This association was characterized as a mechanism of resistance to the KIT tyrosine kinase inhibitor dasatinib in preclinical models [126].

The amplification of CDK4 has been found in more than 50% of cases of MM [115,127]. Treatments with the CDK4/6 inhibitor palbociclib in patient-derived xenografts (PDX) resulted in sustained tumor suppressions for eight weeks [128]. The clinical activity in human patients remains to be elucidated.

Other potential drivers, such as *NF1* and *GNAQ/GNA11* mutations, have been described in 7–22% and 9.5% of patients with MM, respectively. Interestingly, tumors with *NF1* alterations have shown to be more resistant to BRAF inhibitors in preclinical models [129,130,131]. Adequate estimations of the frequency of these mutations cannot be established since multiple studies have reported conflicting results (0–18%) [68,131,132,133].

Finally, mutations in *SF3B1* represent 35% of MMs, most commonly found in anorectal and vulvovaginal localizations [105,134]. While clinical implications of this alteration are still not fully elucidated, a meta-analysis including 53 cases with *SF3B1* mutations suggested a trend to better the OS [115].

### 3.3. Immunotherapy

Before the immune and targeted therapy era, chemotherapy was the unique option for treating patients suffering from advanced MM. In terms of response to cytotoxic chemotherapy, MM patients exhibited a limited efficacy, similarly to CM. Single-agent or combined regimens showed responses between 15% and 25%, respectively, but without further improved survival advantages [135,136,137].

On the other hand, immunotherapy has demonstrated to be a more suitable option in MM than chemotherapy (Table 3). A French multicenter retrospective study compared immunotherapy (*n* = 151) vs. chemotherapy (*n* = 78) as treatment strategies for stage IIIC-IV MM [138]. The authors found a significantly longer median OS for patients in the immunotherapy (anti-PD-1 and anti-CTLA-4) group (15.97 months) as compared to those receiving chemotherapy, mainly dacarbazine (8.82 months).

Concomitantly, a post-hoc analysis of pembrolizumab in 84 patients with advanced mucosal melanoma of KEYNOTE-001, -002, -006 showed an ORR of 22% (95% CI 11–35%) and 15% (95% CI 5–32%) in ipilimumab-naive and ipilimumab-treated patients, respectively. The median PFS in the entire cohort was 2.8 months (95% CI 2.7% to 2.8%) [139].

Notably, anti-PD-1 seems to be associated with a higher efficacy than anti-CTLA-4 in MM, as it was demonstrated in a cohort of 44 first-line-treated patients with unresectable and/or metastatic MM. Patients achieved an ORR of 35% and a median PFS of 5 months using pembrolizumab compared to an ORR of 8.2% and a PFS of 5 months in the ipilimumab group [140].

As a result of the impressive efficacy achieved by combining anti-CTLA-4 and anti-PD-1 in CM, this strategy was analyzed for MM. In a pooled analysis of six trials (phases 1, 2, and 3), including 157 patients with MM, D’Angelo et al. compared nivolumab plus ipilimumab, nivolumab alone, and ipilimumab alone [141]. As expected, the combined regimen achieved a better ORR (37.1%), as compared to nivolumab or ipilimumab monotherapies (23.3% and 8.3%, respectively).

Similarly, combined immunotherapy was evaluated in a subgroup of treatment-naive stage III or IV MM patients treated in CheckMate 067 with nivolumab plus ipilimumab (*n* = 28), nivolumab (*n* = 23), or ipilimumab (*n* = 28). Better outcomes were found for patients receiving the combination (ORR 43% and PFS 5.8 months), as compared to nivolumab (ORR 30% and PFS 3 months) and ipilimumab (ORR 7% and PFS 2.6 months) [142]. 

Altogether, these findings support the idea that immunotherapy is a valuable treatment option for MM. The combination of anti-PD-1 and anti-CTLA-4, seems to be a rational strategy for the initial treatment approach, despite having a high incidence of toxicity (55% of grade 3 to 4 adverse events) [11]. However, the ORR is still lower than CM, which is probably explained by the lower TMB observed in this subtype [116,143]. 

Remarkably, a more recent strategy may enhance the efficacy of immunotherapy. Angiogenesis modulates the tumor microenvironment of different tumors, including melanomas, and vascular endothelial growth factor (VEGF) was proposed to playing an immunosuppressive role [144,145]. This rationale, already tested in renal cell carcinoma with unprecedented results, was evaluated in two-phase 1b and 2 MM trials combining toripalimab, a recombinant humanized PD-1 monoclonal antibody, with the VEGF-receptor inhibitors axitinib or vorolanib. Both studies showed encouraging results (Table 3) [146,147,148,149]. The phase 1b trial conducted by Sheng et al. investigated the combination of toripalimab and axitinib in 29 treatment-naive patients with metastatic MM [148]. This study showed impressive responses and disease control rates of 48.5% and 84.8%, respectively. The median PFS and OS were 7.5 and 20.7 months, respectively. Of note, no significant differences were observed according to the PD-L1 expression or TMB. It should be highlighted that although these results need to be validated in larger studies, this combination represents one of the most effective strategies for the treatment of advanced MM to date.

## 4. Uveal Melanoma

### 4.1. Introduction

Uveal melanoma (UM) is the most common primary intraocular malignancy in adults, representing ~5% of all melanomas [2]. Although UM can arise from the pigmented tissue of the iris and ciliary body, more than 90% of cases emerge from choroids [153]. In contrast to the increasing rate of CM cases observed in the last years, the incidence of UM has remained relatively stable at approximately five per million since the 1970s [154]. This subtype is especially prevalent among white patients with light-colored eyes [155].

Of note, the typical UV mutational signature has not been identified in UM since the cornea, lens, and vitreous act as a barrier between most UV radiation and the choroids [156]. As a consequence, UM shows a remarkably low mutational burden, except for iris melanomas that have been associated with UV-induced DNA damage.

As expected, visual disorders are the most common symptom. However, almost one-third of cases are incidentally detected in a routine ophthalmologic exam [157]. Otherwise, treatment approaches are oriented to preserve eye and vision and include phototherapy, plaque brachytherapy, photon stereotactic radiation therapy, local resection, and enucleation for locally advanced cases. Of note, the metastatic pattern of UM is quite distinctive, characterized by hematogenous dissemination. Liver involvement may occur in approximately 50% of patients within the first 5 years following diagnosis, or even up to 25 years later [158]. In this scenario, liver-directed therapy is a commonly selected strategy, including surgery, chemoembolization, radioembolization, immunoembolization, and the hepatic arterial infusion of chemotherapy [159].

### 4.2. Genetic Landscape and Targeted Therapy Approaches

#### 4.2.1. Gα_q_ Signaling

UMs are not characterized by targetable mutations in *BRAF*, *NRAS, or KIT* (Figure 1). Instead, they show a specific somatic mutation profile characterized by oncogenic mutually-exclusive mutations in either *GNAQ*, *GNA11*, or sporadically in *PLCB4* or *CYSLTR2* genes [160]. These mutations lead to Gα_q_ pathway activation with the subsequent stimulation of the MAPK and β-catenin pathways, as well as the transcriptional co-activator Yes-associated protein 1 (YAP1) through the Trio-Rho/Rac signaling circuit [161,162].

Mutations in *GNAQ* and *GNA11* genes are considered an early development event and are present in ~85% of all UMs [161,163,164]. Hotspot *GNAQ* p.Q209 mutations are found in 45% of primary UM and 22% of metastases, while *GNA11* p.Q209 mutations are found in 32% of primary tumors and 57% of UM metastases [165]. Consequently, it was proposed that *GNA11* mutations have a more relevant effect on tumorigenesis since *GNA11* Q209 mutations are more frequently observed in the metastasis of UM. Additionally, in mouse models, *GNA11* mutations demonstrated to be more tumorigenic than *GNAQ* mutations [166]. Less frequently, a second mutation was also described at codon p.R183 in both genes (6%).

CYSLTR2-mediated signaling promotes the activation of a variety of downstream pathways, including PKC, MAPK, and PI3K signaling. The p.Leu129Gln substitution of *CYSLTR2* produces a constitutive activation of endogenous Gα_q_ and can promote tumorigenesis in vivo [167]. *CYSLTR2* somatic mutations were found in around 4% of UM.

The PLCB4 (phospholipase C β4) protein plays a crucial role in the intracellular transduction of extracellular signals in the retina and is another downstream effector of Gα_q_ signaling. A gain-of-function mutation of this gene was reported at a low frequency in UM (2.5%) [13,167]. *PLCB4* p.D630Y mutations are mutually exclusive with mutations in *GNAQ/GNA11*.

Concerning the systemic treatment, chemotherapeutic regimens are often recommended in CM, such as dacarbazine, cisplatin, and temozolomide, which were evaluated in patients with UM and poor ORRs (<10%) were observed [168].

Notably, other strategies were developed, taking into account that the typical mutations in *GNAQ*/*GNA11* in UM lead to constitutive activation of the MAPK and PI3K/AKT pathways. Thus, logical approaches considered downstream targeted therapies against effector proteins, such as MEK and AKT. Some clinical trials were developed based on this rationale of inhibition of downstream Gα_q_, (Table 4). In this context, selumetinib (an oral selective MEK1/2 inhibitor) was tested against chemotherapy (temozolomide or dacarbazine) in a phase 2 trial, and in combination with dacarbazine in the phase 3, multicenter, and randomized SUMIT trial. Unfortunately, both studies showed limited clinical activity (ORR 14% and 3%, respectively) in advanced UM patients [169,170]. Subsequently, the MEK inhibition trametinib was tested alone or in combination with the AKT inhibitor GSK2141795 in a phase 2 trial, including patients with advanced UM [171]. The combination did not improve the clinical outcomes since patients in the trametinib arm (*n* = 18) achieved an ORR of 5.5% compared to 4.8% in the combined arm (*n* = 21). The median PFS was 3.6 months in both groups.

Based on the concept that UMs normally synthesize and secrete vascular endothelial growth factor (VEGF), an additional targeted therapy tested was the oral multi-kinase inhibitor sunitinib [181]. Scheulen et al. developed a phase 2 trial recruiting 118 chemonaive patients with metastatic UM. Unfortunately, only two cases had a partial response (1.7%), 78 had a stable disease (66.1%), and the median PFS was 5.5 months [178].

Although the rationale for all these targeted therapies was innovative, the clinical efficacy is still disappointing.

#### 4.2.2. Others

The bi-allelic inactivation of the tumor suppressor gene BAP1 (BRCA1-associated protein 1), accounting for 60% of UMs, is another critical genetic alteration for UM development. This mutation was related to the metastatic relapse pattern and worse outcomes [182]. *BAP1* germline mutations were also linked to a hereditary predisposition to UM [183]. Of note, UM can appear in the context of the *BAP1*-tumor predisposition syndrome, which is associated with an increased risk for skin cancer (CM and basal cell carcinoma), renal cell carcinoma, and malignant mesothelioma [184].

Together with *BAP1* mutations, *SF3B1* (splicing factor 3b subunit 1), and *EIF1AX* (eukaryotic translation initiation factor 1A, X-linked) formed a second mutually exclusive subgroup in UM. Mutations in *SF3B1*, most commonly in amino acid 625 (R625), were reported in approximately 10–21% of UM cases [185,186,187]. The prognosis of these mutations has conflicting data. Harbour et al. found that patients with *SF3B1*-mutated UM had a better prognosis compared with the *SF3B1* wild-type patients, while Yavuzyigitoglu et al. found that *SF3B1* mutations were associated with a significantly worse prognosis and the development of late metastasis [188,189]. Otherwise, *EIF1AX-*mutant UM occurs in ~20% of UM cases, and a complete understanding of the functional effects of this mutation remains unknown. Notably, *EIF1AX-*mutant patients showed a better prognosis [186,189].

Furthermore, UM involves additional molecular alterations, such as chromosomal aberration losses of 3, 1p, 6q, 8p and 16q, and the amplification of chromosome arms 6p and 8q. Based on the multiplatform analysis of 80 primary UMs, The Cancer Genome Atlas (TCGA) project helped to categorize this rare disease into four main groups with different genetic and immunological profiles, molecular alterations, and prognoses [190,191]. In summary, groups A and B harbor *EIF1AX* and *SF3B1* mutations and have a more favorable prognosis, while groups C and D are characterized by *BAP1* mutations and worse outcomes.

Supported by the role of BAP1 in DNA damage repair, an interesting phase 2 trial would be to explore the efficacy of the PARP inhibitor niraparib in several tumors, including UM, harboring *BAP1* and other DNA damage response mutations (NCT03207347) [192].

### 4.3. Tumor Immunogenicity and Therapy

The eye may provide a protective environment for UM development and growth. As a consequence, UM can evade immune surveillance via multiple mechanisms, including a deficiency of co-stimulatory molecules in the presentation of antigens process (CD80 and CD86), and by producing immunosuppressive cytokines, such as IDO1 [190,193,194,195,196]. In the same context, PDL-1 has been reported in around 5.1% of metastatic UMs and 10.6% of primary samples [97,197]. Interestingly, after analyzing 80 primary samples, Basile and colleagues described that PDL-1 was inversely correlated with the tumor thickness, PFS, and OS [198]. Authors have also highlighted the role of HLA-G and certain inmune-checkpoint related genes, such as CD47, CD200, TNFRSF6B, HVEM, and GAL9 as predictive factors for disease-free survival. Furthermore, it was also reported that the leukocyte fraction of tumor immune infiltrates is very low in UM, and only group D is considered inflammated [190,191,194].

Under these circumstances, the role of immunotherapy in this population is still unclear, since the immune checkpoint inhibitors as a monotherapy (anti-CTLA-4 or anti-PD-1/PD-L1) demonstrated a restricted activity in small conducted studies (ORR 0–7% and median OS < 1 year (Table 4)). A phase 2 trial developed by The Dermatologic Cooperative Oncology Group (DeCOG), which investigated the efficacy of ipilimumab 3mg/kg among 53 patients with treatment-naive metastatic UM. The disease control rate was 47%, and no patients had a complete or partial response. The progression-free survival and OS were 2.8 and 6.8 months, respectively [174]. Similarly, Tsai et al. evaluated pembrolizumab, nivolumab, and atezolizumab as monotherapies in a multicenter retrospective study in 58 metastatic UM. The overall response rate, PFS, and OS were 3%, 2.7 months, and 9.5 months, respectively [176].

On the other hand, Piulats et al. showed the most promising results to the date with immunotherapy. In a phase 2 single-arm trial, the authors evaluated the combination of ipilimumab and nivolumab in patients with metastatic UM in a first-line setting. Among the 19 cases enrolled, the ORR was 15.8%, and disease stabilization was achieved in 47.4% of patients. With a median follow-up of 4.6 months, the median PFS was 4.99 months, and the median OS was not reached [177].

Despite the limited results with regard to immune checkpoint inhibition, other novel immune-based approaches were investigated. Encouraging preliminary activities were observed with the novel bispecific protein tebentafusp (IMCgp100) which targets a fragment of the melanocytes lineage-specific antigen gp100 in the context of HLA-A2 (50% of Caucasians) [199,200]. The phase 1/2 study enrolled 19 patients with metastatic UM, and despite having a considerable immune-related toxicity profile, a tumor response was observed in 31.6% of patients, and the one year PFS and OS rates were 66% and 74%, respectively [180]. The pivotal phase II study of IMCgp100 is ongoing (NCT03070392) [201]. Another promising strategy is glembatumumab vedotinb, a monoclonal antibody-drug conjugate against the a transmembrane glycoprotein gpNMB, overexpressed by multiple tumor types, including MM. In the phase 2 trial, including 31 patients with advanced UM, the ORR was 6% and the DCR was 61%, while the median PFS and OS were 3.2 months and 11.8 months, respectively [179].

Taking into account all of these studies to date, the treatment of advanced UM remains a challenge since the impressive results obtained in CMs could not be translated to this rare subtype.

## 5. Unusual or Unknown Primary Site Melanomas

Unusual primary sites of melanoma (UPSM) are defined as those which do not arise from the skin, the uveal tract of the eye, or various mucosal surfaces. On the other hand, the melanoma of an unknown primary (MUP) is defined as the histologically confirmed presence of melanoma in the lymph nodes, viscera, or distant skin subcutaneous tissue, without a history of primary melanoma. An adequate characterization of UPSMs and MUPs is subject to controversy, considering that some cases of MUPs cannot be sharply distinguished from UPSM. Our approach is to consider UPSMs as unique entities, represented by a single lesion that arises in uncommon localizations. These types of entities, such as dermal melanomas and melanomas arising in brain and meninges, are usually associated with clinical and biological characteristics that support this distinction. Furthermore, CM with spontaneous remission is a potential differential diagnosis that has to be taken into consideration. In this way, some classic studies have excluded MUP characterizations—cases where patients reported a history of skin lesion resection or eye enucleation [202].

According to the different case series and reviews published, the incidence of MUPs is between 2% and 5% of all melanomas. MUPs are classified into three categories, including subcutaneous, nodal, and visceral diseases [1]. Nodal is usually considered the most common subtype ranging between 0.7% and 8.8%, being axillar lymph nodes among the most frequent initial localizations [203]. Nevertheless, it needs to be highlighted that the definition of MUP varies among series, and a large Dutch retrospective study, which gathered information from 2028 patients with MUP, has described that a visceral involvement can be evidenced in around 51% of included patients [203].

A systematic review and meta-analysis provided evidence that MUP is associated with a better prognosis than stage III (HR 0.83, 95% CI 0.73–0.96) and stage IV CM (HR 0.85, 95% CI 0.75–0.96). The role of a spontaneous immune response in the primary lesion may be defined as an interesting explanation for this finding [204]. Interestingly, the genetic characterization of this entity showed a higher rate of mutations in *BRAF* and *NRAS* genes, representing 53% and 14% of analyzed samples respectively, as well as more mutations in the *TERT* promoter [205].

Recent studies have intriguingly suggested that patients with MUP may benefit from immunotherapy. Gambicher et al., described that seven of nine patients (77.8%) diagnosed with MUP achieved disease control after immunotherapy initiation [206]. In the Dutch series, Verver and colleagues compared the outcomes of MUP patients that were treated with novel therapies, such as checkpoint inhibitors or targeted therapies, with the other group of patients included in the registry from 2011 to 2016 [203]. The authors reported that the median OS was 11 and 4 months, respectively. Notably, the included patients that had undergone targeted therapies and immune checkpoint inhibitors in the first-line treatment experienced a median OS of 18 months. These results suggest that the definition of treatment in MUP should follow the current treatment strategies for CM.

Nowadays, MUP and UPSM are a diagnosis of exclusion, and efforts should be oriented to perform sufficient procedures to exclude the potential primary localizations. Initial strategies include clinical examination, imaging diagnosis, otorhinolaryngological, ophthalmological, gynecological, and urological exams when appropriate. Anorectal, subungual, and mucosal inspections are necessary examinations to a complete assessment, which also may include lower and upper gastrointestinal, bronchial, and nasopharyngeal endoscopies. Positron emission tomography (PET), with 18F-fluoro-2-deoxy-d-glucose (FDG), is superior to morphologic imaging to detect systemic disease and has replaced MRI, CT, and exhaustive endoscopies as the first steps of examination. A meta-analysis that included 2150 patients with advanced-stage melanomas (III and IV) found that FDG-PET had a sensitivity of 86% and 87% specificity for detecting metastases [207].

The identification of radiopharmaceutical tracers is a priority to better characterize rare tumors. Emerging models include the combination of monitoring fatty acid uptake and lipoprotein lipase with desorption electrospray ionization-mass spectrometry (DESI-mS), dual-isotope theranostics with fluorine-18 and DOTA, ^18^F-labeled benzamide, gold nanoparticles conjugated with the α-melanocyte-stimulating hormone peptide radiolabeled with ^64^Cu, and iodinated melanin-targeting compounds [208]. The activity of these methods for tracing melanoma in human patients is yet to be confirmed.

### 5.1. Brain and Meninges

Primary intracranial and meningeal melanomas (PIMMs) are rare tumors probably derived from neural crest cells. PIMM represents 1% of all melanomas and 0.05% of primary brain tumors. The main areas involved are the pons, cerebellum, cerebral peduncles, interpeduncular fossa, base brain, and spinal cord (cervical region) [209].

The World Health Organization (WHO) classification divides the melanocytic lesions of the central nervous system into three types: diffuse melanosis, meningeal melanocytoma (benign course), and melanoma [210]. Considering the latter, two forms of PIMM should be highlighted: solid tumors and diffuse meningeal melanomatosis [211].

A diagnostic workup usually involves an MRI scan and biopsy or cytology of suspicious lesions. MRI characteristic lesions usually exhibit hyper-intensity on T1 and iso- to hypo-intensity on T2. Immunohistochemistry is crucial and recommended in pathologic analyses. PIMMs are commonly positive for HMB-45, melan A, and S-100 [212]. This technique is especially useful for detecting amelanotic meningeal melanoma (AMM), representing 10% of PIMMs [213].

The median OS of patients with a PIMM diagnosis is around one year. Of note, a better prognosis was reported in patients with primary spinal cord lesions [214].

PIMM is associated with a low immunogenicity. These tumors have similar mutations to UM, including a high prevalence of *GNAQ/GNA11* mutations, and infrequent *BRAF* mutations. Kusters et al. reported the first whole-exome sequencing analysis of a primary leptomeningeal melanoma, showing a total of 27 somatic mutations, which accounts for a relatively low number in comparison to other melanoma subtypes [215].

Although there is no standard treatment for primary PIMMs, surgery is probably the main local control approach, followed by stereotaxic or whole-brain radiotherapy as an adjuvant treatment in selected cases.

Results of modern therapies for PIMMs have been scarcely reported. Fujimori et al. described one of the first case reports of a patient with *BRAF*-mutant PIMM treated with vemurafenib and nivolumab, with no clinical benefit [216]. El Habnouni et al. reported a case of an 86-year-old female with a PIMM that harbored a *BAP1*-inactivating mutation and undetectable PDL-1, with treatment failure after 16 weeks of pembrolizumab [217]. The primary resistance to modern therapies could be explained by the indemnity of the blood–brain barrier in patients with PIMM.

### 5.2. Primary Dermal Melanoma

Primary or solitary dermal melanomas (PDM) are typically described as well-circumscribed nodules localized in the dermis or subcutaneous tissue, without any evidence of involvement or signs of tumor regression in the epidermis.

Cassarino and colleagues’ analysis of 13 samples of PDM supports the existence of particular immunohistochemical characteristics that may distinguish between primary nodular melanoma, metastatic cutaneous melanoma, and PDM [218]. According to the authors’ findings, the latter is associated with a low expression of cyclin D1, Ki-67, D2-40 and p53, which can explain the less aggressive behavior of this tumor model. Teow and collaborators did not find evidence of BRAF mutations in a series of 12 cases of PDM [219].

A lymph node compromise is rarely observed in PDM. In a series reported by Harris et al., none of the 32 patients that underwent a sentinel lymph node biopsy had positive nodes [220]. The five-year OS in 62 patients was 87.1%, supporting the already reported favorable prognosis of patients with this particular subtype [221]. Consequently, wide excisions, with 2-cm margins are usually the selected treatment approach. Due to its infrequency, a sentinel node biopsy is not uniformly conducted in this population [219].

### 5.3. Esophageal Melanoma

The occurrence of melanoma in the esophagus in classically explained by the presence of melanocytes in up to 2.5–8% of the tissue, especially in the middle and lower third of the organ [222]. Schizas and colleagues have recently reported an exhaustive systematic review that collected data from 93 patients with esophageal melanoma reported in 59 studies [223]. The authors reported an increased incidence of male patients (2:1) having tumors most frequently localized at the lower (48.4%) and middle esophagus (46.2%). Multifocality and necrosis were observed in approximately one in five cases.

Surgical treatments should be carefully planned, and usually consist of a total or subtotal esophagectomy or a gastrectomy in the cases where the gastroesophageal junction is involved. Mainly, lymph node involvement can be found in up to 66% of patients with esophageal melanoma [224]. An extended lymphadenectomy is often performed as part of a surgical treatment in this setting, usually including mediastinal, esophageal, and celiac axis nodes. Nevertheless, Schizas and colleagues did not find a statistically significant association of lymphadenectomy and OS [223]. The prognosis of primary esophageal melanoma is poor. Gao and colleagues have reported in a series of 17 patients a 5-year OS of 10%, and a median OS of only 18.1 months [225].

There are only a few reports regarding the use of checkpoint inhibitors in advanced settings. Rochefort and colleagues published a 75-year-old patient who received treatment with nivolumab, achieving disease stability for approximately six months before progression [225]. In their series, Hashimoto et al. included a case of a patient that only received two cycles of nivolumab before clinical deterioration. Due to its rarity, a standardized treatment for this disease has not been established [226].

### 5.4. Primary Malignant Melanoma of the Breast and other UPSM

Primary malignant melanomas of the breast (PMMB) are exceedingly rare entities [227]. There are differential diagnoses, such as poorly differentiated breast carcinomas, hystiocytic sarcomas, and clear cell sarcomas.

In this particular setting, the distinction between a PMMB and a MUP is particularly challenging. In fact, a classical series of the MD Anderson Cancer Center documented that melanoma is the most frequent origin of metastases in the breast, accounting for 38.5% of the 169 evaluated cases [228]. While the characterization of PMMB has been limited to a few literature reports, Rassouli and colleagues explained that PMMB might arise from ectopic melanocytes incorporated in the breast epithelium, or as the result of metaplastic changes in breast duct cells [229]. In their literature review, only two of the evaluated cases presented axillary lymph node metastases, which could be interpreted as a distinctive metastatic pattern of PMMB.

Standard treatments for this localization cannot be defined, and a common approach includes surgical resection and a sentinel lymph node biopsy. An adjuvant radiotherapy can be considered in selected scenarios. Notably, the case reported by Rassouli et al., and the two patients documented by Koh and colleagues, were associated with *BRAF* V600E mutations [230].

The characterization of other UPSM is challenging. Uncommon locations for UPSMs, such as lung, esophagus, pancreas, bone, heart, spleen, and lymph nodes, have been reported in the literature as case reports or series, hampering an adequate characterization [231]. The surgical treatment represents a reasonable strategy for isolated UPSMs.

Given the limited information in the literature, the treatments for patients with UPMS are often defined on a case by case basis. Nevertheless, anatomic criteria should also be considered in the definition and characterization of rare melanomas, and more efforts are needed to understand the pathogenesis and biological particularities of these infrequent subtypes.

## 6. Conclusions

Rare diseases are often defined as entities with a low incidence. Our approach was to determine clinical–biological characteristics and treatment perspectives regarding uncommon melanoma subtypes with distinctive anatomical, histological, or molecular features.

In recent years, impressive progress has been achieved for CM, particularly combining surgery and high-efficacy new drugs. Even in distinctive subgroups not prospectively evaluated in phase 3 randomized clinical trials, such as CM with NRAS mutations, treatment strategies typically include surgical approaches for localized melanoma and immune checkpoint inhibitors in advanced scenarios.

The advent of targeted therapies highlights the role of molecular characterization in melanomas. BRAF V600 mutations are identified mostly in CM but should also be considered in the treatment algorithm of MM and even in atypical tumor localizations, such as PMMB. Emerging agents, directed to other possible actionable mutations, support the necessity of the further comprehensive molecular profiling of the different melanoma models addressed in this review. Interestingly, the identification of ALK isoforms, and ROS1 or NTRK fusions, may bring new treatment options for CM patients.

The identification of specific mutation signatures, such as UV-induced signatures, may also contribute to treatment decisions. This mutational pattern may explain the high response rates reported in patients with desmoplastic melanoma after immune checkpoint inhibitors. Notably, a substantial benefit with immunotherapy was also documented in patients with angiosarcoma of the head, neck, face, and scalp when UV-induced mutations were noticed [232].

Interestingly, recent clinical trials evaluating MM have shown stimulating results using immunotherapy, especially in combination, and with tyrosine kinase inhibitors. However, clinical benefits are still limited when compared to CM. Similarly, both therapies have shown a restricted efficacy for other melanomas subtypes, such as UM.

Our review also included the analysis of UPSM, considering that these extremely infrequent entities may harbor distinctive clinical and biological characteristics, including specific mutations, spreading routes, and prognoses. The current literature supports this categorization for some atypical localizations, such as the case of the high prevalence of GNAQ/GNA11 mutations in PIMMs, or the favorable prognosis and infrequent lymph node involvement evidenced in patients with PDMs.

Other entities initially localized in atypical organs, such as the breast, lung, kidney, or liver, represent a truly diagnostic challenge, and current evidence does not allow a clear separation between a UPSM and a MUP. In these cases, physical exams and further workups to identify a more typical primary site is the first essential step.

Possibly, in this scenario, genomic tumor profiling represents an opportunity to improve diagnoses and clinical management. In this context, the NOMINATOR study documented the results of the next-generation sequencing profiling of 121 patients with rare cancers [233]. An actionable finding was evidenced in 51% of the included cases, and notably, four patients with an original diagnosis of soft tissue tumors were genotypically recategorized as NF1 and high TMB melanomas with UV-induced mutations.

Consequently, more efforts should be made to characterize the molecular patterns and to define treatment perspectives for patients with rare melanomas, including primaries with uncommon localizations and MUP. Importantly, a multidisciplinary approach remains crucial to address and guide patient care in patients with these rare conditions.

## Figures and Tables

**Table 1:** Selected NRAS-mutant melanoma clinical trials.

Study	Phase	*N*	Arms	ORR (%)	DCR (%)	PFS (mo)	OS (mo)
Dummer et al. 2017 [18]	3 ^a,e^	402	Binimetinib; Dacarbazine	15; 7	58; 25	2.8; 1.5	11; 10.1
Lebbe et al. 2016 [19]	2 ^b,e^	194	Pimasertib; Dacarbazine	27; 14	33; 16	3.3; 1.7	8.9; 10.6
Ascierto et al. 2013 [20]	2 ^d^	30	Binimetinib	10	63	3.7	NS
Kirkwood et al. 2012 [21]	2 ^c,e^	10; 18	Selumetinib; Temozolomide	0; 6	50; 55	NS	NS
Kim et al. 2019 [22]	1 ^e^	9	Belvarafenib	44	NS	6.2	NS
Schuller et al. 2017 [23]	1b ^e^	16	Ribociclib + Binimetinib	25	69	6.7	NS
Algazi et al. 2017 [24]	1 ^d,e^	10	GSK2141795 ^f^ + Trametinib	0	40	2.3	4
Sullivan et al. 2017 [25]	1 ^e^	18	Ulixertinib	17	NS	NS	NS
Falchook et al. 2012 [26]	1 ^b,e^	7	Trametinib	0	22	NS	NS

Abbreviations: ORR, overall response rate; DCR, disease control rate; PFS, progression-free survival; OS, overall survival; NS, not specified. ^a^ Analysis of patients with cutaneous melanoma or melanoma with an unknown primary; ^b^ analysis of patients with cutaneous melanoma; ^c^ analysis of patients with cutaneous and mucosal melanoma or melanoma with an unknown primary; ^d^ analysis of patients with cutaneous and mucosal melanoma.; ^e^ results shown only for patients with *NRAS* mutations; ^f^ AKT inhibitor.

**Table 2:** Selected clinical trials that assessed KIT inhibitors in melanoma.

Study	Phase	*N*	Subtype (n)	Arms	ORR (%)	DCR (%)	PFS (mo)	OS (mo)
Kim et al. 2008 [86]	2 ^b^	21	Cutaneous (7) Acral (2) Soft part (1) Unclassified (11)	Imatinib	4.8	23.8	1.4	7.5
Guo et al. 2011 [87]	2 ^a^	43	Acral (21) Mucosal (11) Cutanous (9) Unknown (2)	Imatinib	23.3	53.5	3.5	14
Carvajal et al. 2011 [88]	2^a^	28	Mucosal (13) Acral (10) Cutaneous (5)	Imatinib	Mucosal 23 Acral 38 Cutaneous 0	NS	2.8	10.7
Hodi et al. 2013 [89]	2 ^a^	24	Mucosal (17) Acral (6) Cutenous (1)	Imatinib	29	50	3.7	12.5
Cho et al. 2012 [90]	2 ^a^	11	Acral (9) Mucosal (2)	Nilotinib	22.2	77.8	2.5	7.7
Carvajal et al. 2015 [91]	2 ^a,e^	19	Mucosal (12) Acral (4) Cutaneous (3)	Nilotinib	Mucosal 27.2 Acral 0 Cutaneous 0	Mucosal 63.6 Acral 25 Cutaneous 33.3	3.4 ^f^ 2.6 ^g^	14.2 ^f^ 4.3 ^g^
Lee et al. 2015 [92]	2 ^b^	27	Acral (15) Mucosal (7) Cutaneous (5)	Nilotinib	Acral 40 Mucosal 0 Cutaneous 0	Acral 73.3 Mucosal 28.6 Cutaneous 40	NS	NS
Guo et al. 2017 [93]	2 ^b^	42	Acral (20) Mucosal (20) Cutaneous (2)	Nilotinib	Acral 25 Mucosal 25 Cutaneous 50	Acral 80 Mucosal 70 Cutaneous 50	4.2	18
Deylon et al. 2018 [94]	2 ^b^	22	Mucosal (9) Acral (7) Cutaneous (6)	Nilotinib	Mucosal 33.3 Acral 14.3 Cutaneous 16.6	Mucosal 66.6 Acral 71.4 Cutaneous 80	6 ^d^	13.2 ^d^
Kalinsky et al. 2016 [95]	2 ^b^	25	Acral (15) Mucosa (10)	Dasatinib	Acral 33 Mucosal 14	50 ^c^	2.7	11.8
Minor et al. 2012 [96]	- ^b^	6	Mucosal (NS) Acral (NS) Cutaneous (NS)	Sunitinib	Mucosal 60 Acral 0	Mucosal 60 Acral 0	NS	NS

Abbreviations: ORR, overall response rate; DCR, disease control rate; PFS, progression-free survival; OS, overall survival; NS, not specified. ^a^ Included patients with KIT amplifications; ^b^ only patients with KIT mutations; ^c^ considering 22 patients in part II with KIT mutations; ^d^ included three patients with KIT amplifications; ^e^ after prior treatment with imatinib; ^f^ patients without central nervous system metastases; ^g^ patients with central nervous system metastases.

**Table 3:** Selected studies that assessed immunotherapy in advanced mucosal melanoma.

Study	Study Type	*N*	Arms (n)	ORR (%)	DCR (%)	PFS (mo)	OS (mo)
Postow et al. 2013 [150]	Multicenter, retrospective	33	Ipilimumab	6.7	26.7	NS	6.4
Del Vecchio et al. 2014 [151]	Expanded, access program	71	Ipilimumab	11	36.2	4.3	6.4
Shoushtari et al. 2016 [100]	Multi-institutional, retrospective	35	Nivolumab or Pembrolizumab	23	42.9	3.9	NS
D’Angelo et al. 2017 [141]	Pooled analysis of phase 1-2-3 studies ^b^	157	Nivolumab + Ipilimumab (86) Nivolumab (35) Ipilimumab (36)	37.1 23.3 8.3	57.1 45.3 16.7	5.9 3 2.7	NS
Mignard et al. 2018 [138]	Multicenter, retrospective	151	Ipilimumab (76) Nivolumab or Pembrolizumab (75)	11.9	17.9	15.97	NS
Omid et al. 2019 [139]	Post-hoc analysis of phase 1-2-3 studies ^a^	84	Pembrolizumab	19	31	2.8	11.3
Moya-Plana et al. 2019 [140]	Single-center prospective cohort	44	Ipilimumab (24) Pembrolizumab (20)	8.2 35	30 45	3 5	12 16.2
Si Lu et al. 2019 [152]	Phase 1b	15	Pembrolizumab	13.3	20	NS	NS
Shoushtari et al. 2020 [142]	Subgroup of CheckMate 067	79	Ipilimumab + Nivolumab (28) Nivolumab (23) Ipilimumab (28)	43 30 7	57 39 11	5.8 3.0 2.6	22.7 20.2 12.1
Sheng et al. 2020 [148]	Phase 1b	29	Axitinib + Toripalimab	48.5	84.8	7.5	20.7
Si Lu et al. 2020 [149]	Phase 2	40	Vorolanib + Toripalimab	15–22.2	55.5–65	5.6–5.7	NS

Abbreviations: ORR, overall response rate; DCR, disease control rate; PFS, progression-free survival; OS, overall survival; NS, not specified. ^a^ Post-hoc analysis of KEYNOTE-001, -002, -006 (phase 1, 2, and 3, respectively); ^b^ pooled analysis of phase 1 CA209-003, phase 1 CA209-038, phase 3 CheckMate 066, phase 3 CheckMate 037; phase 3 CheckMate 067, phase 2 CheckMate 069.

**Table 4:** Selected studies for therapy of uveal melanoma.

Study	Study Type	*N*	Arms (n)	ORR (%)	DCR (%)	PFS (mo)	OS (mo)
Luke et al. 2013 [172]	Multicenter, retrospective	39	Ipilimumab	2.6	46	-	9.6
Piulats et al. 2014 [173]	Phase 2	32	Ipilimumab	6.45	50	NS	NS
Carvajal et al. 2014 [169]	Phase 2	101	Selumetinib (50) Chemotherapy (51)	14 0	NS	3.7 1.6	11.8 9.1
Zimmer et al. 2015 [174]	Phase 2	53	Ipilimumab	0	47	2.8	6.8
Joshua et al. 2015 [175]	Phase 2	11	Tremelimumab	0	-	2.9	12.8
Shoushtari et al. 2016 [171]	Phase 2	39	Trametinib (18) Trametinib + GSK2141795 ^a^ (21)	5.5 4.8	NS	3.6 3.6	NS
Tsai et al. 2016 [176]	Multicenter, retrospective	58	Pembrolizumab (40) Nivolumab (16) Atezolizumab (2)	3	10	2.7	9.5
Piulats et al. 2017 [177]	Phase 2	19	Nivolumab + Ipilimumab	15.8	63.2	4.99	NR
Scheulen et al. 2017 [178]	Phase 2	118	Sorafenib	1.7	66.1	5.5	14.8
Patel et al. 2017 [179]	Phase 2	31	Glembatumumab Vedotin ^b^	6	61	3.2	11.8
Mignard et al. 2018 [138]	Multicenter, Retrospective	100	Ipilimumab (63) Nivolumab or Pembrolizumab (37)	0	32	-	13.38
Carvajal et al. 2018 [170]	Phase 3	129	Selumetinib + Dacarbazine (97) Placebo + Dacarbazine (32)	3 0	NS	2.8 1.8	NS
Sato et al. 2018 [180]	Phase 1/2	19	Tebentafusp ^c^	10.5 ^d^	-	-	NR

Abbreviations: ORR, overall response rate; DCR, disease control rate; PFS, progression-free survival; OS, overall survival; NS, not specified; NR, not reached. ^a^ AKT inhibitor; ^b^ monoclonal antibody-drug conjugate against NMB; ^c^ bispecific protein IMCgp100; ^d^ minor responses in 4 patients.
